# Understanding the relationships between trauma type and individual posttraumatic stress symptoms: a cross‐sectional study of a clinical sample of children and adolescents

**DOI:** 10.1111/jcpp.13602

**Published:** 2022-03-18

**Authors:** Marianne Skogbrott Birkeland, Ane‐Marthe Solheim Skar, Tine K. Jensen

**Affiliations:** ^1^ Norwegian Centre for Violence and Traumatic Stress Studies Oslo Norway; ^2^ 25566 Department of Psychology University of Oslo Oslo Norway

**Keywords:** Trauma, posttraumatic stress disorder

## Abstract

**Background:**

Characteristics of traumatic events may be associated with the level and specific manifestation of posttraumatic stress symptoms (PTSS). This study examined the differences and similarities between overall levels, profiles and networks of PTSS after sexual trauma, domestic violence, community violence, non‐interpersonal trauma, sudden loss or serious illness of a loved one, and severe bullying or threats.

**Methods:**

PTSS were measured in a clinical sample of 4,921 children and adolescents (6–18 years old, *M* = 14.0, *SD* = 2.7, 63.7% female) referred to Child and Adolescent Mental Health Services. We compared 95% confidence intervals (CI) for each symptom with 95% CI for overall PTSS within each trauma type (self‐reported worst trauma). We also computed cross‐sectional networks and searched for differences in networks according to trauma type and overall symptom level.

**Results:**

The overall frequencies of PTSS were highest following sexual trauma; somewhat lower for domestic violence and severe bullying or threats and lowest after community violence, non‐interpersonal trauma and sudden loss or serious illness. Psychological cue reactivity, avoidance and difficulties with sleeping and concentrating were generally among the most frequent symptoms. Sexual trauma, domestic violence and severe bullying or threats were associated with higher frequencies of negative beliefs and persistent negative emotional states. Few differences in symptom networks across trauma type emerged.

**Conclusion:**

Different types of trauma exposure may be associated with different profiles of symptom frequencies. Knowledge about this may be useful for clinicians and for the movement towards evidence‐based personalized psychological treatment.

## Introduction

Traumatic events differ in severity, duration and meaning and may be associated with specific profiles of posttraumatic stress symptoms (PTSS) in youth. A clearer understanding of the profiles of PTSS after exposure to different types of trauma may help us to design effective personalized interventions that prevent long‐term consequences.

According to the DSM‐5, there are four clusters of PTSS: intrusion symptoms; avoidance of internal and external trauma reminders; negative alterations in cognitions and mood and alterations in arousal and reactivity (American Psychiatric Association, 2013). Some types of trauma have been found to be associated with more severe overall PTSS. For example, survivors repeated interpersonal trauma show higher levels of PTSS than survivors of single‐event traumas (Ehring & Quack, 2010). Along the same lines, sexual trauma has been found to be associated with higher levels of PTSS than non‐sexual domestic or community violence (Smith, Summers, Dillon, & Cougle, 2016).

Such broad classifications may, however, mask differences in symptom profiles associated with characteristics of each trauma type (Grimm, Hulse, Preiss, & Schmidt, 2012). For example, motor vehicle accidents and non‐sexual domestic and community trauma have been found to be associated with higher levels of symptoms within the intrusion and the hyperarousal clusters (Kelley, Weathers, McDevitt‐Murphy, Eakin, & Flood, 2009; Smith et al., 2016). For sudden loss, symptoms within avoidance and negative alterations in cognitions and mood seem to be more common (Kelley et al., 2009). Regarding sexual trauma, different studies have pointed to higher symptom levels within all four clusters (Forbes et al., 2014; Kelley et al., 2009; Smith et al., 2016). While these studies examined levels of symptoms across trauma type, they did not explore whether certain symptoms are more prominent for any particular trauma type. For example, if sexual trauma is associated with higher levels of intrusion symptoms compared to domestic violence, this could reflect the generally higher levels of symptoms after sexual trauma rather than indicating that intrusion symptoms are particularly dominant after sexual trauma. Therefore, to acquire knowledge about the most dominant symptoms, it is necessary to compare symptom levels both *across* and *within* each trauma type.

To help us understand why different trauma types may be associated with different symptom profiles, we use the predictive processing perspective. According to this perspective, people use past experiences to predict future events with the greatest possible precision (Kube, Berg, Kleim, & Herzog, 2020). Based on ambiguous evidence, people form hypotheses about the threats in the world. For example, based on one’s experiences with men, one might evaluate the hypothesis ‘Some men are dangerous’ as more probable than the competing hypothesis ‘All men are dangerous’. These hypotheses are predictions that govern how people interact with the world. Building on Terr’s classification of childhood trauma into types 1 and 2 (Terr, 1991), Wilkinson, Dodgson, and Meares (2017) argue that the two types are associated with different information processing and different hypotheses or predictions about the world. Type 1 trauma is single traumatic events that are usually life threatening (e.g. motor vehicle accident), whereas type 2 trauma occurs over an extended period of time (e.g. domestic violence). For experiences that are perceived as life threatening (type 1), it is important not to miss the cues and to avoid similar experiences in order to survive. Therefore, such events can give rise to hypotheses given an unusually – and possibly unrealistically – high probability of being true. For example, after experiencing a single motor vehicle accident, the hypothesis ‘Generally, I am in danger when I am in a car’ may be evaluated as having an 80% probability of being true and selected over ‘Generally, I am *not* in danger when I am in a car’ (20% true), even though the fit with the overall input from the environment is relatively poor. Importantly, the choice of the less probable hypothesis does not generalize to other threats. Thus, according to this theory, being in a car accident would not lead to a general hypothesis that ‘The world is a dangerous place’. Rather, the hypothesis is specific and connected to cues that signal danger (e.g. cars).

Type 2 trauma, however, involves repeated experiences over an extended period of time that may give the impression that the world is actually not kind or safe. According to the predictive processing perspective, this leads to biased information processing that is generally biased towards threatening hypotheses, rather than the very specific hypothesis seen with type 1 trauma (Wilkinson et al., 2017). For example, exposure to domestic violence may skew all hypotheses about oneself, others and the world towards the negative end of the scale. As a result, domestic violence could lead to the selection of the more threatening hypotheses in many situations: for example, ‘I have no value’, ‘Others cannot be trusted’ and ‘Bad things will always happen to me’. Specific hypotheses about possible dangers connected to cues (associated with type 1 trauma) may be followed by symptoms of intrusion, avoidance and alterations in arousal and reactivity. With generally biased information processing leading to many threatening hypotheses (associated with type 2 trauma), the symptomatology may be more general and more often involve negative alterations in cognitions and mood.

From the predictive processing perspective, we assume that some posttraumatic symptoms may be more connected to others, dependent upon trauma types. For example, after motor vehicle accidents, symptoms involved in the fear conditioning circuit may be particularly strongly connected. Network analyses are one way to examine this, but as far as we know, no previous studies have compared different profiles of PTSS in children and adolescents after different trauma experiences.

Overall, studies suggest that different types of trauma may be associated with different levels of PTSS in children and youth. However, it remains unclear what types of trauma may be associated with which profiles and networks of PTSS. Therefore, we investigated the following research questions:

1. *Are different types of trauma associated with different profiles (i.e. different descriptions of the frequencies of all individual PTSS) of posttraumatic stress symptoms in youth?* We hypothesize that type 1 trauma (e.g. accidents, natural disasters, medical trauma, community violence) is associated with high levels of intrusions, avoidance and alterations in arousal and reactivity, whereas type 2 trauma (e.g. sexual trauma, domestic violence, severe bullying or threats from peers) is associated with high levels of negative alterations in cognitions and mood. Some traumatic experiences may have features of both type 1 and type 2. Sudden loss or serious illness of a loved one is a single event, but in line with Terr (1991), we acknowledge that such experiences have permanent or long‐lasting effects on children’s lives that may be followed by considerable changes in how they think and feel about themselves, others and the world. Thus, sudden loss and serious illness of a loved one may be more difficult to place into only one of the categories, and we hypothesize that this type of trauma may be followed by a flatter profile of symptoms than the other trauma types.
2. *Are different types of trauma associated with different cross‐sectional networks (i.e. systems of interconnection between the individual PTSS) of posttraumatic stress symptoms in youth?* We hypothesize that negative beliefs have a particularly strong role after type 2 trauma and that symptoms of re‐experiencing, hyperarousal and avoidance are particularly strongly connected to each other after type 1 trauma.

## Method

### Participants and procedure

This is a cross‐sectional study of children and youth referred to the Norwegian Child and Adolescent Mental Services between 2015 and 2017. All referred children were screened for trauma exposure at intake, and those reporting at least one potentially traumatic experience were screened for PTSS. The study was approved by the Norwegian Regional Committee for Medical and Health Research Ethics, and a waiver of consent was granted because the data obtained were de‐identified.

In total, 18,546 children and youth (55.1% girls, *M* age = 12.9, *SD* age = 3.2) were screened for trauma exposure. The checklist for traumatic experiences was introduced by ‘The questions below describe different experiences that children and youth can have. Please indicate if you have experienced some of the following events, and you felt scared, confused or helpless’. At the end of the checklist, the participants were asked to indicate their worst traumatic event: ‘Which of these events bother you the most at the current time point?’ (see Table S1). The inclusion criteria for the current study were as follows: children who reported at least one traumatic event (*n* = 14,248, 77%, 57.5% girls, *M* age = 13.2, *SD* age = 3.1) and were screened for PTSS according to DSM‐5 (*n* = 4,925, 63.7% girls, *M* age = 14.0, *SD* age = 2.7). We excluded those screened for PTSS according to DSM‐IV, and those below the age of 6 years or over the age of 18 years. This provided us with a total sample of *n* = 4,921 (see Table 1).

**Table 1:** Descriptive data of the sample

	Total sample (*n* = 4,921) % (*n*)/mean (*SD*)	Included sample (*n* = 2,387) % (*n*)/mean (*SD*)
Female sex	63.7 (3,134)	65.8 (1,502)
Age	14.0 (2.7)	14.0 (2.7)
Number of traumatic events	3.7 (2.3)	3.1 (2.1)
Sum of PTSS	20.7 (14.0)	19.7 (13.7)
Probable PTSD	47.6 (2,342)	44.4 (1,061)

Included sample includes respondents who reported their worst trauma types to be either sexual trauma, domestic violence, community violence, non‐interpersonal trauma, sudden loss or serious illness of a loved one, or severe bullying or threats.

### Types of traumatic events

Participants had been exposed to 1–15 traumatic events (*M* = 3.7, *SD* = 2.3). For those with multiple exposures, we created mutually exclusive categories of youth, based on what they indicated was their worst traumatic experience. Of the 4,921 screened for PTSS, 2,761 participants reported one worst traumatic experience, 529 reported two types of worst traumatic experiences, 213 reported three or more types of worst experiences and 1,418 did not report any types of worst traumatic experience. The worst reported trauma types were categorized as follows: sexual trauma (‘touching of private parts’ and/or ‘rape’, *n* = 350); domestic violence (‘witnessing violence in the family’ and/or being ‘exposed to physical violence in the family’, *n* = 402); community violence (‘assault, robbery, violence or threats of violence outside the family’ and/or ‘witnessing violence outside the family’, *n* = 98); non‐interpersonal trauma (‘severe accident’, ‘natural disaster’ and/or ‘medical trauma’, *n* = 237); sudden loss or serious illness of a loved one (*n* = 650) and severe bullying or threats (*n* = 650). Very few participants (<1%) reported terrorism or war (*n* = 4), abduction (*n* = 5) or someone taking pictures of their private parts (*n* = 5) as their worst trauma, and those who did were not included in this categorization. As we did not know what experiences the category of ‘other frightening experiences’ (*n* = 108) included, those who indicated this was their worst experience were not included in this categorization. The group of children reporting other frightening experiences than those in the trauma checklist as their worst traumatic experiences seem to be similar to those included in sample in terms of demographics, but reported lower levels of PTSS (see Table S1).

In addition, we excluded participants who had experienced more than one traumatic experience without reporting which was the worst, participants who reported three or more worst traumatic experiences, and participants who reported two worst traumatic experiences of different types (e.g. sexual trauma and severe bullying/threats). Thus, 2,387 participants were included in the trauma type analyses. Compared to the 2,534 participants not included, a significantly lower percentage of the 2,387 included participants reported PTSS above the clinical threshold (44.4% vs. 50.6%, *p* = <.001).

### Measurements

#### Posttraumatic stress symptoms (PTSS)

Posttraumatic stress symptoms (PTSS) were measured using the child and adolescent trauma screen (CATS; Sachser et al., 2017). This is a self‐report questionnaire developed for children and adolescents, measuring PTSD symptoms according to the DSM‐5. The 20 items map directly onto criteria B (intrusions), C (avoidance), D (negative alterations in cognitions and mood) and E (alterations in arousal and reactivity). Symptom frequency for the previous 2 weeks was rated using a 4‐point scale: from 0 (*Not at all*) to 3 (*5 or more times a week/almost always*). In cases where the children reported a score between the response categories, scores were rounded up. The total sum scores range from 0 to 60. A sum score of 21 or above is considered to indicate probable PTSD (University of Ulm, 2017). The CATS has demonstrated convergent validity and internal consistency (Sachser et al., 2017). In the current study, Cronbach’s alpha was .93.

### Missing data

Of the 4,921 participants, 9.1% (*n* = 450) had missing data for age and 5.1% (*n* = 250) had missing data for gender. The percentage of missing data at the item level for PTSS items varied between 0.8% (*n* = 37, nightmare item) and 4.6% (*n* = 227, detachment item). For 78.7% of the sample (*n* = 3,871), we had data for age, gender and all the PTSS items. In summary, we had data for 96.7% of the 104,658 possible data points for these items. Sum/average scores were computed if the participant had responded to at least half of the items of the scale (Fairclough, 2010, p. 50).

### Statistical analyses

To assess frequencies and profiles of PTSS across worst trauma type, we conducted an ANOVA with Scheffe post hoc tests comparing overall PTSS across worst trauma type, as well as calculated means and 95% CI for each of the symptoms (1,000 bootstraps).

To examine associations between the individual PTSS, we conducted cross‐sectional network analyses. We estimated a regularized partial correlation network and applied the extended Bayesian information criterion (EBIC) graphical lasso. We performed robustness analyses by assessing the variability of edge weights and centrality by estimating CI within which the true value of the parameter lies in 95% of cases (bootstrapped samples = 1,000). Network stability can be quantified using the correlation stability (CS) coefficient, which should not be below .25 and preferably be above .50 (Epskamp, Borsboom, & Fried, 2018).

To detect significant differences in the networks across trauma type, we used an approach constructed to recursively split the sample based on covariates. To take differences in mean levels into account when searching for differences in network across trauma exposure and avoid invoking Berkson’s bias (Haslbeck, Ryan, & Dablander, 2021), we searched for splits in networks based on both (a) trauma exposure only and (b) on trauma exposure and mean levels of symptoms combined. In this way, we were able. The analyses were conducted in SPSS (IBM Corp, 2019) and R – specifically the packages qgraph (Epskamp, Cramer, Waldorp, Schmittmann, & Borsboom, 2012), bootnet 1.4.3 (Epskamp & Fried, 2018) and networktree (Jones, Mair, Simon, & Zeileis, 2020).

## Results

### Trauma types and profiles of posttraumatic stress symptoms (PTSS)

The overall level of PTSS varied across worst trauma type. The youth reporting sexual trauma had a higher PTSS level than all other trauma types (*M* = 28.8, *SD* = 13.4, 70.6% probable PTSD, all *p*s < .001). The youth reporting severe bullying or threats (*M* = 21.2, *SD* = 12.6, 50.6% probable PTSD) and the youth reporting domestic violence (*M* = 21.0, *SD* = 13.7, 49.0% probable PTSD) had symptoms of a higher level than the other trauma types (all *p*s < .001). However, the overage levels of symptoms of the youth reporting domestic violence, and the youth reporting severe bullying or threats, were not significantly different from each other (*p* = 1.000). The youth reporting sudden loss or serious illness of a loved one (*M* = 15.7, *SD* = 12.5, 31.7% probable PTSD), community violence (*M* = 14.6, *SD* = 12.6, 28.6% probable PTSD) or non‐interpersonal trauma (*M* = 12.6, *SD* = 11.7, 22.8% probable PTSD) did not display significantly different levels of overall PTSS (*p* = .079 to .988).

**Figure 1:**
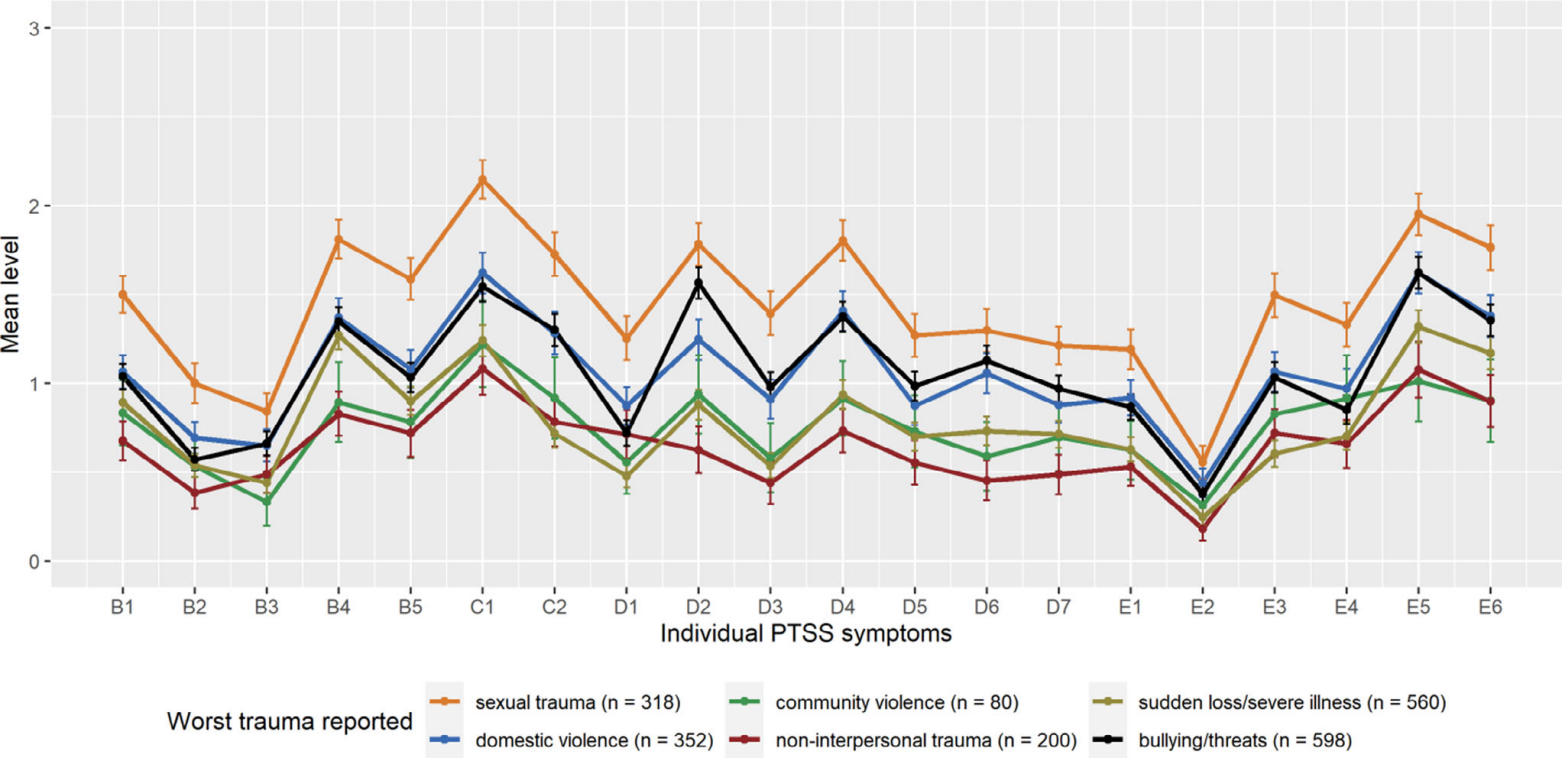
Means with 95% CI of each of the individual PTSS symptoms according to worst trauma reported. Note: B1: recurrent thoughts of trauma, B2: recurrent dreams of trauma, B3: flashbacks, B4: psychological cue reactivity, B5: physiological cue reactivity, C1: avoidance of thoughts of trauma, C2: avoidance of reminders of trauma, D1: memory impairment, D2: negative beliefs, D3: distorted blame, D4: persistent negative emotional state, D5: diminished interested in activities, D6: feelings of detachment from others, D7: inability to experience positive emotions, E1: irritability or anger, E2: reckless/self‐destructive behaviour, E3: hypervigilance, E4: exaggerated startle response, E5: difficulty concentrating, E6: sleeping difficulties

Figure 1 presents means and 95% CI of each of the symptoms according to the worst trauma reported (see Tables S2 and S3 for details). Across all assessed worst traumatic experiences, the most commonly endorsed symptoms were psychological cue reactivity, avoidance and difficulties concentrating and sleeping. Of special interest may be that the symptom profile for youth exposed to severe bullying or threats was similar to the symptom profile for youth exposed to domestic violence. One exception is that youth exposed to severe bullying or threats reported higher frequencies of negative beliefs than did youth exposed to domestic violence.

In addition, Figure 1 shows that sexual trauma and severe bullying or threats were also associated with higher frequencies of negative beliefs and negative emotions, compared to many other symptoms. Thus, the CI of these individual symptoms were not overlapping with those of the mean symptoms within the respective trauma type. Domestic violence and sudden loss or serious illness were associated with relatively high frequency of negative emotions.

The different profiles of PTSS may be associated with cumulative exposure to trauma types rather than the trauma type itself. Additional analyses of means and 95% CI of each of the symptoms according to the number of trauma types (see Tables S4 and S5) show that the same symptoms were dominant regardless of number of trauma types reported. These findings indicate that this interpretation is less likely.

### Trauma types and networks of posttraumatic stress symptoms (PTSS)

**Figure 2:**
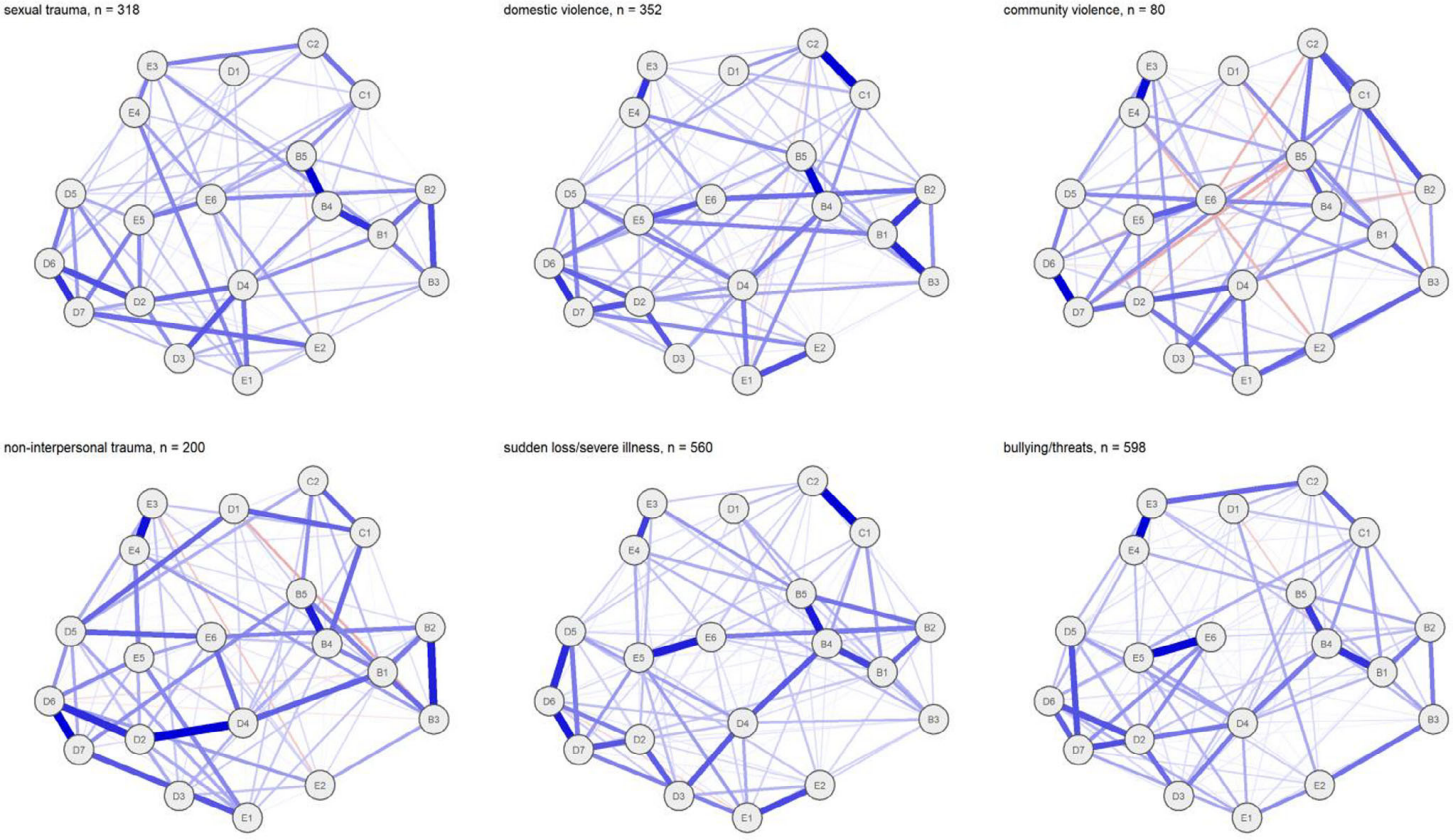
Networks of PTSS, according to worst trauma reported. B1: recurrent thoughts of trauma. B2: recurrent dreams of trauma. B3: flashbacks. B4: psychological cue reactivity. B5: physiological cue reactivity. C1: avoidance of thoughts of trauma. C2: avoidance of reminders of trauma. D1: memory impairment. D2: negative beliefs. D3: distorted blame. D4: persistent negative emotional state. D5: diminished interested in activities. D6: feelings of detachment from others. D7: inability to experience positive emotions. E1: irritability or anger. E2: reckless/self‐destructive behaviour. E3: hypervigilance. E4: exaggerated startle response. E5: difficulty concentrating. E6: sleeping difficulties

We calculated networks for each of the trauma types (see Figure 2). Before interpreting the networks, we calculated the CS coefficients. CS coefficients above .25 and preferably above .50 indicate sufficient stability for interpretation. The CS coefficients for *edges* (the connections between each of the symptoms) for each of the networks were as follows: sexual trauma: .67; domestic violence: .59, community violence: .36; non‐interpersonal trauma: .44; sudden loss or severe illness: .67; and severe bullying or threats: .67. This indicates that the networks have sufficient stability for interpretation, with some caution required for community violence and non‐interpersonal trauma.

The CS coefficients for *strength centrality* were as follows: sexual trauma: .67; domestic violence: .67; community violence: .05; non‐interpersonal trauma: .44; sudden loss or severe illness: .75 and severe bullying or threats: .75. This indicates that values for strength centrality were relatively stable and can be interpreted for sexual trauma, domestic violence, non‐interpersonal trauma, sudden loss or severe illness and severe bullying or threats, but not for community violence.

Persistent negative emotional state and negative beliefs seem to have consistently high strength centrality across the trauma types (see Figure S1). In addition, psychological cue reactivity and physiological cue reactivity seem to be of high centrality.

When searching for splits in network based on the six trauma types, we found the sexual trauma network to be significantly different from the others. The three main differences were that, for those exposed to sexual trauma, the associations between irritability or anger and exaggerated startle response and between flashbacks and distorted blame were stronger, whereas the association between physiological cue reactivity and reckless or self‐destructive behaviour was weaker. However, when we searched for splits based on both trauma exposure and mean levels, all detected splits were based on mean levels. This indicates that the main differences in networks were associated with differences in mean levels of symptoms rather than trauma type.

## Discussion

In this study, we set out to assess whether different types of trauma are associated with different profiles and networks of posttraumatic stress symptoms in youth. The findings are threefold. First, the most pronounced differences across trauma types seem to be in the level of PTSS. Sexual trauma, domestic violence and severe bullying or threats were associated with the highest mean levels of PTSS, whereas community violence, non‐interpersonal trauma and sudden loss or serious illness were associated with lower levels of PTSS. Second, symptoms within the intrusion cluster (psychological cue reactivity), avoidance cluster (both internal and external) and alterations in arousal and reactivity (difficulties with concentration and sleep) were the most common symptoms across trauma type. In addition, symptoms within the negative alterations in cognitions and mood cluster were more dominant than other symptoms after sexual trauma, severe bullying or threats, domestic violence and sudden loss or severe illness of a loved one. Third, the networks of PTSS were similar across trauma exposure.

The finding that sexual trauma was associated with the highest levels of PTSS is in line with the literature (Kelley et al., 2009; Smith et al., 2016). The current study expands the literature by demonstrating that exposure to severe bullying or threats seem to be followed by a similar level of PTSS as domestic violence. This may be interpreted in the light of the increasing importance of peer acceptance during adolescence (Brown & Larson, 2009). Therefore, and in line with other researchers (Bjornsson et al., 2020; Idsoe et al., 2021), we argue that severe and threatening acts of bullying is a type of interpersonal trauma that needs to be considered within the trauma literature.

The most dominant individual PTSS across trauma type were psychological cue reactivity, avoidance (internal and external) and difficulties with concentrating and sleeping. Children and youth may be particularly impacted by problems with concentration and sleeping as it interrupts the ability to learn in school. Psychological cue reactivity and avoidance are symptoms closely related to fear conditioning. That these symptoms emerged as generally prominent suggests that fear conditioning may be an important part of PTSS in youth.

Sexual trauma, domestic violence and severe bullying/threats were associated with particularly high frequencies of negative beliefs and persistent negative emotional states, which is consistent with the predictive processing perspective on PTSS – namely, that type 2 trauma might have particularly severe consequences for youths’ general information processing. According to the predictive processing perspective on PTSS (Wilkinson et al., 2017), such events teach victims to choose threatening hypotheses over more optimistic ones, thus causing them to experience negative beliefs and mood. By predicting that ‘If I trust other people, I will get hurt’ rather than ‘If I trust other people, I will get help if I need it’, one might feel more protected and less exposed, based on a ‘better safe than sorry’ philosophy. Thus, type 2 trauma may have more severe consequences for general expectations for the future, and victims may be more suspicious of other people. Consistent with this, studies have found that maltreatment, domestic violence and sexual trauma may be associated with greater attentiveness to potential threats (Briggs‐Gowan et al., 2015), higher levels of distrust and slower learning of trust in behavioural tasks (Hepp, Schmitz, Urbild, Zauner, & Niedtfeld, 2021). This may have severe consequences for future development. For example, maltreated children appear to follow two general developmental pathways: withdrawal from others or heightened aggression towards others (Cicchetti, 2016). Thus, this negatively biased predictive processing may contribute to explaining why type 2 trauma may have severe consequences into adulthood.

We found no clear support for the hypothesis that fear conditioning symptoms are more prominent after type 1 trauma (non‐interpersonal). This might be because the general symptom levels were quite low, and larger sample size might be needed to detect the differences between symptoms.

The network analyses revealed that two pairs of symptoms were more tightly connected with each other after sexual trauma than after other trauma: (a) exaggerated startle response and irritability or anger and (b) flashbacks and distorted blame. As we found no evidence for differences in networks when we accounted for overall levels of symptoms, we cannot conclude whether this is specific to sexual trauma or if this is a feature of PTSS that emerges for particularly high levels of PTSS. However, the main differences in symptom presentation may not lie in the connections between the symptoms, but in the PTSS profile. For some people, however, symptoms may influence each other over time, in a self‐reinforcing loop, regardless of the event that started it, and ultimately turn into PTSD. This would be in line with dynamic systems theory, which conceptualizes the transition into disorder as a shift between two states; and according to network theory, this may be dependent of network connectivity (Borsboom et al., 2016). In other words, low‐to‐moderate levels of PTSS may be conceptualized as a continuum, but in individuals with high levels of symptoms and high connectivity between them, symptoms may transition into a discrete, qualitatively different state. Once the symptoms have formed a tightly connected network and PTSD has emerged, the trauma type might be of less importance.

Our results may also be interpreted as supporting the view that interpersonal trauma increases the probability of displaying not only re‐experiencing, avoidance and hyperarousal, but also a set of other symptoms related to disturbances in self‐organization, such as affective dysregulation, negative self‐concept and disturbances in relationships. Moreover, some children exposed to non‐interpersonal traumas also reported negative cognitions and emotions. In sum, these findings lend support to the theoretical rationale for complex PTSD (Cloitre, Garvert, Brewin, Bryant, & Maercker, 2013; Elliott et al., 2021). Experiencing trauma in childhood or adolescence may disturb the development of healthy interpersonal skills, self‐identity and affect regulation.

Among the strengths of this study is the large size of the clinical sample. Data were collected in similar ways across trauma type and sophisticated statistical methodology was used. However, the cross‐sectional design prohibited conclusions about direction or causality between symptoms. Although the youth were asked to specify their worst experience, many were multi‐traumatized, and the symptoms may be the result of their total trauma history. In addition, we have no data on the pervasiveness of the traumatic exposure (e.g. the number of times exposed to sexual trauma or the duration of domestic violence) or the time elapsed since the traumatic events, which means that some of the symptoms may be stronger because they are closer in time to the moment of measurement. There may also be large variation in the specific experiences within each of trauma types. We were not able to explore potential differences related to this. We did not use a measure that specifies the concrete acts that may constitute severe and threatening bullying. We did not investigate potentially different symptom manifestations across age and gender. We used a measure of traumatic exposure that mentioned the A2 criterion (subjective emotional response) from DSM‐IV. As potentially traumatic events were measured retrospectively, they may be biased by current PTSS. We had no data on sociodemographic characteristics of the sample. It is worth noticing that the results are based on a clinical treatment‐seeking sample and may not be generalized to the general population or to youth with PTSD.

In conclusion, the different types of trauma exposure may be associated with different profiles of symptom frequencies. Sexual trauma, domestic violence and severe bullying or threats were associated with more negative beliefs. Sudden loss and severe illness were associated with negative emotions. From a developmental psychological perspective, it is crucial that we understand the consequences of the traumatic event for each individual child or adolescent so that we can counteract them and lead the youth into a positive developmental trajectory. Future studies that investigate relationships between profiles of PTSS and factors such as timing since trauma and cumulative exposure may further benefit our understanding of development of specific symptoms.

The results of the current study may have implications for responsiveness to treatment interventions and may enable us to take the next steps towards personalized medicine. For example, interventions that target negative beliefs (e.g. cognitive restructuring) may be particularly potent for adolescents who report sexual trauma, domestic violence or severe bullying or threats as their worst traumatic experience. Since sleeping and concentration difficulties are significant symptoms across all trauma types, therapists should pay particular attention to these symptoms. For PTSS, there are several well‐documented evidence‐based treatment options, and now there is a need for more research on how to adapt them to individuals and design effective personalized interventions that prevent long‐term consequences for each individual.

## Supporting information


**Figure S1.** Strength centrality of each of the individuals PTSS symptoms according to worst trauma reported.
**Table S1.** Descriptive data of the included sample compared to those reporting other frightening experiences as their worst trauma type.
**Table S2.** Exposure to potential traumatic events within each of the six groups based on worst trauma reported.
**Table S3.** 95% CI of each of the individual PTSS symptoms according to worst trauma reported.
**Table S4.** Exposure to potentially traumatic events, according to number of trauma types reported.
**Table S5.** 95% CI of each of the individual PTSS symptoms according to number of trauma types reported.
