# Supplementary material for: Understanding the relationships between trauma type and individual posttraumatic stress symptoms: a cross‐sectional study of a clinical sample of children and adolescents
Source: J Child Psychol Psychiatry. 2022 Mar 18;63(12):1496–504. doi: 10.1111/jcpp.13602 (PMC9790300; doi:10.1111/jcpp.13602)
Supplement: Supplementary file 1 — Figure S1. Strength centrality of each of the individuals PTSS symptoms according to worst trauma reported. Table S1. Descriptive data of the included sample compared to those reporting other frightening experiences as their worst trauma type. Table S2. Exposure to potential traumatic events within each of the six groups based on worst trauma reported. Table S3. 95% CI of each of the individual PTSS symptoms according to worst trauma reported. Table S4. Exposure to potentially traumatic events, according to number of trauma types reported. Table S5. 95% CI of each of the individual PTSS symptoms according to number of trauma types reported. [file JCPP-63-1496-s001.docx]

Supporting Information

**Understanding the relationships between trauma type and individual posttraumatic stress symptoms: A cross-sectional study of a clinical sample of children and adolescents**

Marianne Skogbrott Birkeland^1^

Ane-Marthe Solheim Skar^1^

Tine K. Jensen^1, 2^

^1^Norwegian Centre for Violence and Traumatic Stress Studies, Norway

^2^Department of Psychology, University of Oslo, Norway

* Corresponding author, [Marianne.s.birkeland@gmail.com](mailto:Marianne.s.birkelang@gmail.com)

Table S1. Descriptive data of the included sample compared to those reporting other frightening experiences as their worst trauma type

|  | **Included sample (n = 2,387)**  **% (n) / mean (SD)** | **Other frightening experience^1^ (n = 108)**  **% (n) / mean (SD)** | **Test of difference** |
| --- | --- | --- | --- |
| Female sex | 65.8 (1,502) | 62.4 (63) | Fischer’s exact test = 0.45, p = .266 |
| Age | 14.0 (2.7) | 13.8 (2.8) | F = 0.35, p = .553 |
| Sum of PTSS | 19.7 (13.7) | 15.8 (13.3) | F = 8.842, p = .004 |
| Probable PTSD | 44.4 (1,061) | 32.4 (35) | Fischer’s exact test = 0.01, p = .008 |

^1^Participants who reported “other frightening experiences” than the 14 specific events in the checklist in S1, as their worst trauma type

Table S2. Exposure to potential traumatic events within each of the six groups based on worst trauma reported

|  | **Sexual trauma**  **(n = 350)** | **Domestic violence**  **(n = 402)** | **Community violence**  **(n = 98)** | **Non-interpersonal trauma**  **(n = 237)** | **Sudden loss/ serious illess**  **(n = 650)** | **Severe bullying/threats (n = 650)** |
| --- | --- | --- | --- | --- | --- | --- |
| Severe accident | 16.0 (n = 56) | 17.9 (n = 72) | 17.7 (n = 17) | 46.4 (n = 110) | 12.3 (n = 80) | 12.5 (n = 81) |
| Natural disaster | 11.7 (n = 41) | 11.2 (n = 45) | 9.2 (n = 9) | 16.0 (n = 38) | 10.0 (n = 65) | 9.5 (n = 62) |
| Terrorism/war | 3.7 (n = 13) | 2.7 (n = 11) | 5.1 (n = 5) | 1.7 (n = 4) | 5.1 (n = 33) | 1.5 (n = 10) |
| Medical | 24.6 (n = 86) | 25.6 (n = 103) | 22.4 (n = 22) | 58.2 (n = 138) | 20.0 (n = 130) | 21.7 (n = 141) |
| Sudden loss/serious illness of loved one | 53.1 (n = 186) | 38.3 (n = 154) | 30.6 (n = 30) | 30.8 (n = 73) | 100.0 (n = 650) | 39.7 (n = 258) |
| Severe bullying/threats | 52.0 (n = 182) | 40.0 (n =161) | 41.8 (n = 41) | 23.2 (n = 55) | 27.7 (n = 180) | 100 (n = 650) |
| Abduction | 8.0 (n = 28) | 6.0 (n = 24) | 2.0 (n = 2) | 3.0 ( n = 7) | 4.0 (n = 26) | 2.9 (n = 19) |
| Exposed to physical violence outside family | 32.3 (n = 113) | 19.4 (n = 78) | 72.4 (n = 71) | 12.2 (n = 29) | 15.2 (n = 99) | 32.8 (n = 213) |
| Witnessed physical violence outside family | 28.9 (n = 101) | 25.6 (n = 103) | 49.0 (n = 48) | 13.1 (n = 31) | 19.7 (n = 128) | 22.0 (n = 143) |
| Witnessed physical violence inside family | 24.0 (n = 84) | 62.2 (n = 250) | 12.2 (n = 12) | 5.9 (n = 14) | 11.4 (n = 74) | 9.4 (n = 61) |
| Exposed physical violence inside family | 31.4 (n = 110) | 81.3 (n = 327) | 14.3 (n = 14) | 5.5 (n = 13) | 13.8 (n = 90) | 12.8 (n = 83) |
| Someone taken pictures of private parts | 11.4 (n = 40) | 3.7 (n = 15) | 3.1 (n = 3) | 1.3 (n = 3) | 2.0 (n = 13) | 2.6 (n = 17) |
| Someone touched private parts or forced to touched other’s | 82.0 (n = 287) | 8.7 (n = 35) | 11.2 (n = 11) | 3.4 (n = 8) | 6.8 (n = 44) | 7.7(n = 50) |
| Rape | 61.7 (n = 216) | 5.2 (n = 21) | 2.0 (n = 2) | 1.3 (n = 3) | 2.9 (n = 19) | 2.3 (n = 15) |
| Other | 27.1 (n = 95) | 24.1 (n = 97) | 8.2 (n = 8) | 14.3 (n = 34) | 16.2 (n = 105) | 16.3 (n = 109) |

Table S3. 95% CI of each of the individual PTSS symptoms according to worst trauma reported

|  | **Sexual trauma** | **Domestic violence** | **Community violence** | **Non-interpersonal trauma** | **Sudden loss/serious illness** | **Severe bullying/threats** | **All** |
| --- | --- | --- | --- | --- | --- | --- | --- |
| N | 318 | 352 | 80 | 200 | 560 | 598 | 4274 |
| Mean | 1.38 – 1.53 | 1.02 – 1.17 | 0.64 – 0.91 | 0.57 – 0.74 | 0.72 – 0.82 | 1.02 – 1.12 | 1.03 – 1.08 |
| Intrusion | 1.27 – 1.44 | 0.93 – 1.10 | 0.56 – 0.85 | 0.54 – 0.72 | 0.74 – 0.86 | 0.88 – 1.00 | 0.96 – 1.00 |
| B1 | 1.40 – 1.62 | 1.00 – 1.20 | 0.69 – 1.09 | 0.55 – 0.78 | 0.82 – 0.98 | 0.96 – 1.12 | 1.07 – 1.13 |
| B2 | 0.89 – 1.13 | 0.64 – 0.85 | 0.39 – 0.74 | 0.29 – 0.47 | 0.46 – 0.60 | 0.52 – 0.66 | 0.67 – 0.73 |
| B3 | 0.75 – 0.96 | 0.61 – 0.81 | 0.24 – 0.54 | 0.49 – 0.62 | 0.38 – 0.50 | 0.60 – 0.75 | 0.62 – 0.68 |
| B4 | **1.73 – 1.94** | **1.30 – 1.53** | 0.67 – 1.17 | 0.74 – 1.01 | **1.17 – 1.34** | **1.27 – 1.44** | **1.33 – 1.40** |
| B5 | 1.47 – 1.71 | 1.01 – 1.26 | 0.57 – 1.01 | 0.59 – 0.88 | 0.80 – 1.03 | 0.94 – 1.12 | 1.06 – 1.12 |
| Avoidance | **1.85 – 2.06** | **1.37 – 1.59** | **0.93 – 1.35** | **0.83 – 1.10** | **0.88 – 1.03** | **1.36 – 1.52** | **1.35 – 1.42** |
| C1 | **2.06 – 2.28** | **1.53 – 1.78** | **1.06 – 1.59** | **0.96 – 1.27** | **1.12 – 1.31** | **1.46 – 1.64** | **1.53 – 1.60** |
| C2 | **1.60 – 1.86** | **1.18 – 1.43** | 0.71 – 1.16 | 0.65 – 0.95 | 0.61 – 0.78 | **1.22 – 1.41** | **1.17 – 1.24** |
| Cognitions/mood | 1.36 – 1.53 | 0.97 – 1.14 | 0.59 – 0.88 | 0.48 – 0.67 | 0.64 – 0.76 | 1.04 – 1.16 | 1.01 – 1.06 |
| D1 | 1.12 – 1.38 | 0.77 – 0.99 | 0.39 – 0.80 | 0.58 – 0.87 | 0.38 – 0.51 | 0.64 – 0.79 | 0.77 – 0.83 |
| D2 | **1.68 – 1.92** | 1.14 – 1.39 | 0.70 – 1.18 | 0.50 – 0.79 | 0.76 – 0.95 | **1.46 – 1.64** | **1.27 – 1.34** |
| D3 | 1.31 – 1.57 | 0.83 – 1.05 | 0.42 – 0.85 | 0.33 – 0.58 | 0.46 – 0.61 | 0.88 – 1.06 | 0.88 – 0.95 |
| D4 | **1.71 – 1.95** | **1.34 – 1.58** | 0.74 – 1.19 | 0.58 – 0.84 | **0.87 – 1.04** | **1.28 – 1.45** | **1.31 – 1.38** |
| D5 | 1.14 – 1.38 | 0.79 – 1.02 | 0.49 – 0.90 | 0.43 – 0.69 | 0.61 – 0.77 | 0.90 – 1.07 | 0.91 – 0.98 |
| D6 | 1.19 – 1.44 | 0.95 – 1.18 | 0.40 – 0.81 | 0.34 – 0.57 | 0.63 – 0.80 | 1.05 – 1.23 | 0.98 – 1.04 |
| D7 | 1.12 – 1.32 | 0.79 – 1.01 | 0.50 – 0.91 | 0.36 – 0.58 | 0.72 – 0.78 | 0.88 – 1.04 | 0.89 – 0.96 |
| Arousal/reactivity | 1.29 – 1.46 | 1.00 – 1.14 | 0.60 – 0.90 | 0.58 – 0.76 | 0.71 – 0.83 | 0.96 – 1.06 | 1.01 – 1.05 |
| E1 | 1.08 – 1.32 | 0.82 – 1.04 | 0.40 – 0.71 | 0.43 – 0.65 | 0.55 – 0.70 | 0.80 – 0.95 | 0.85 – 0.91 |
| E2 | 0.48 – 0.67 | 0.37 – 0.53 | 0.18 – 0.45 | 0.12 – 0.26 | 0.20 – 0.30 | 0.31 – 0.42 | 0.36 – 0.41 |
| E3 | 1.38 – 1.64 | 0.97 – 1.20 | 0.57 – 1.05 | 0.55 – 0.83 | 0.53 – 0.68 | 0.96 – 1.14 | 1.01 – 1.07 |
| E4 | 1.18 – 1.44 | 0.86 – 1.08 | 0.63 – 1.15 | 0.51 – 0.78 | 0.62 – 0.79 | 0.77 – 0.93 | 0.92 – 0.99 |
| E5 | **1.82 – 2.06** | **1.52 – 1.76** | 0.81 – 1.31 | **0.91 – 1.23** | **1.19 – 1.39** | **1.52 – 1.71** | **1.53 – 1.61** |
| E6 | **1.60 – 1.87** | **1.24 – 1.48** | 0.66 – 1.13 | 0.72 – 1.23 | **1.05 – 1.25** | **1.24 – 1.43** | **1.31 – 1.39** |

Note: Bold indicates confidence intervals that do not overlap with confidence interval within trauma type, indicating symptoms of higher severity.

B1: recurrent thoughts of trauma.B2: recurrent dreams of trauma. B3: flashbacks. B4: psychological cue reactivity. B5: physiological cue reactivity. C1: avoidance of thoughts of trauma. C2: avoidance of reminders of trauma. D1: memory impairment. D2: negative beliefs. D3: distorted blame. D4: persistent negative emotional state. D5: diminished interested in activities. D6: feelings of detachment from others. D7: inability to experience positive emotions. E1: irritability or anger. E2: reckless/self destructive behaviour. E3: hypervigilance. E4: exaggerated startle response. E5: difficulty concentrating. E6: sleeping difficulties

Table S4. Exposure to potentially traumatic events, according to number of trauma types reported

|  | **1**  **(n = 820)** | **2**  **(n = 953)** | **3**  **(n = 895)** | **4**  **(n = 719)** | **5 or more**  **(n = 1460)** | **All**  **(n = 4921)** |
| --- | --- | --- | --- | --- | --- | --- |
| Severe accident | 4.7 (n = 37) | 10.7 (n = 100) | 18.8 (n = 166) | 19.9 (n = 141) | 35.8 (n = 516) | 20.2 (n = 960) |
| Natural disaster | 1.5 (n = 12) | 6.6 (n = 62) | 8.4 (n = 74) | 14.4 (n = 102) | 27.2 (n = 391) | 13.4 (n = 641) |
| Terrorism/war | 0.5 (n = 4) | 0.8 (n = 8) | 2.4 (n = 21) | 3.9 (n = 28) | 12.7 (n = 185) | 5.1 (n = 246) |
| Medical | 6.8 (n = 54) | 27.8 (n = 165) | 25.7 (n = 226) | 32.6 (n = 231) | 48.7 (n = 699) | 29.0 (n = 1375) |
| Sudden loss/serious illness of loved one | 32.1 (n = 251) | 47.5 (n = 439) | 57.2 (n = 502) | 70.2 (n = 494) | 79.6 (n = 1150) | 59.9 (n = 2836) |
| Severe bullying/threats | 23.5 (n = 184) | 38.5 (n = 355) | 51.2 (n = 444) | 61.5 (n = 432) | 79.3 (n = 1143) | 54.2 (n = 2559) |
| Abduction | 0.6 (n = 5) | 2.1 (n = 20) | 2.5 (n = 22) | 4.5 ( n = 32) | 15.9 (n = 230) | 6.5 (n = 309) |
| Exposed to physical violence outside family | 2.8 (n = 22) | 9.9 (n = 93) | 22.4 (n = 197) | 35.0 (n = 249) | 61.5 (n = 883) | 30.3 (n = 1444) |
| Witnessed physical violence outside family | 1.4 (n = 11) | 8.1 (n = 76) | 20.8 (n = 182) | 30.4 (n = 215) | 58.5 (n = 840) | 27.9 (n = 1324) |
| Witnessed physical violence inside family | 3.0 (n = 24) | 12.7 (n = 119) | 18.8 (n = 165) | 26.2 (n = 186) | 50.0 (n = 718) | 25.5 (n = 1212) |
| Exposed physical violence inside family | 8.0 (n = 64) | 16.8 (n = 152) | 24.2 (n = 212) | 34.7 (n = 245) | 54.8 (n = 787) | 30.7 (n = 1460) |
| Someone taken pictures of private parts | 0.6 (n = 5) | 1.4 (n = 13) | 2.2 (n = 19) | 3.9 (n = 27) | 14.1 (n = 200) | 5.6 (n = 264) |
| Someone touched private parts or forced to touched other’s | 4.1 (n = 33) | 6.8 (n = 64) | 12.9 (n = 112) | 21.2 (n = 148) | 42.3 (n = 602) | 20.3 (n = 959) |
| Rape | 1.1 (n = 9) | 4.1 (n = 38) | 6.7 (n = 58) | 11.7 (n = 81) | 28.7 (n = 401) | 12.6 (n = 591) |
| Other | 14.6 (n = 108) | 23.5 (n = 206) | 35.2 (n = 287) | 41.8 (n = 269) | 58.8 (n = 762) | 37.3 (n = 1632) |

Table S5. 95% CI of each of the individual PTSS symptoms according to number of trauma types reported

|  | **1** | **2** | **3** | **4** | **5 or more** | **All** |
| --- | --- | --- | --- | --- | --- | --- |
| N | 820 | 953 | 895 | 719 | 1460 | 4274 |
| Mean | 0.62 – 0.71 | 0.78 – 0.86 | 0.94 – 1.03 | 1.04 – 1.15 | 1.39 – 1.48 | 1.03 – 1.08 |
| Intrusion | 0.56 – 0.66 | 0.68 – 0.77 | 0.86 – 0.96 | 0.92 – 1.04 | 1.33 – 1.42 | 0.96 – 1.00 |
| B1 | 0.63 – 0.76 | 0.78 – 0.89 | 0.94 – 1.07 | 1.04 – 1.18 | 1.47 – 1.58 | 1.07 – 1.13 |
| B2 | 0.33 – 0.43 | 0.36 – 0.47 | 0.55 – 0.67 | 0.62 – 0.76 | 1.04 – 1.16 | 0.67 – 0.73 |
| B3 | 0.30 – 0.40 | 0.40 – 0.50 | 0.54 – 0.66 | 0.58 – 0.71 | 0.92 – 1.02 | 0.62 – 0.68 |
| B4 | **0.89 – 1.05** | **1.03 – 1.16** | **1.25 – 1.40** | **1.28 – 1.45** | **1.69 – 1.81** | **1.33 – 1.40** |
| B5 | 0.60 – 0.75 | 0.77 – 0.89 | 0.81 – 1.06 | 1.02 – 1.19 | 1.47 – 1.59 | 1.06 – 1.12 |
| Avoidance | **0.87 – 1.02** | **1.06 – 1.19** | **1.26 – 1.40** | **1.38 – 1.55** | **1.72 – 1.83** | **1.35 – 1.42** |
| C1 | **1.01 – 1.17** | **1.22 – 1.37** | **1.46 – 1.63** | **1.54 – 1.73** | **1.91 – 2.03** | **1.53 – 1.60** |
| C2 | 0.71 – 0.88 | **0.89 – 1.04** | 1.03 – 1.20 | **1.20 – 1.40** | **1.51 – 1.64** | **1.17 – 1.24** |
| Cognitions/mood | 0.58 – 0.69 | 0.77 – 0.88 | 0.91 – 1.02 | 1.03 – 1.15 | 1.35 – 1.44 | 1.01 – 1.06 |
| D1 | 0.41 – 0.53 | 0.60 – 0.74 | 0.74 – 0.90 | 0.79 – 0.95 | 0.97 – 1.08 | 0.77 – 0.83 |
| D2 | **0.73 – 0.89** | **0.96 – 1.12** | **1.10 – 1.26** | **1.32 – 1.50** | **1.69 – 1.81** | **1.27 – 1.34** |
| D3 | 0.44 – 0.58 | 0.62 – 0.76 | 0.77 – 0.93 | 0.89 – 1.07 | 1.20 – 1.33 | 0.88 – 0.95 |
| D4 | **0.76 – 0.91** | **1.02 – 1.18** | **1.19 – 1.35** | **1.34 – 1.51** | **1.70 – 1.83** | **1.31 – 1.38** |
| D5 | 0.53 – 0.67 | 0.70 – 0.83 | 0.78 – 0.93 | 0.89 – 1.07 | 1.21 – 1.33 | 0.91 – 0.98 |
| D6 | 0.58 – 0.73 | 0.71 – 0.86 | 0.84 – 0.99 | 0.91 – 1.09 | 1.34 – 1.47 | 0.98 – 1.04 |
| D7 | 0.51 – 0.65 | 0.66 – 0.78 | 0.77 – 0.91 | 0.87 – 1.03 | 1.21 – 1.32 | 0.89 – 0.96 |
| Arousal/reactivity | 0.59 – 0.69 | 0.74 – 0.83 | 0.92 – 1.02 | 1.02 – 1.13 | 1.37 – 1.44 | 1.01 – 1.05 |
| E1 | 0.44 – 0.56 | 0.59 – 0.71 | 0.77 – 0.91 | 0.84 – 0.99 | 1.03 – 1.09 | 0.85 – 0.91 |
| E2 | 0.13 – 0.20 | 0.19 – 0.27 | 0.27 – 0.36 | 0.33 – 0.44 | 0.60 – 0.71 | 0.36 – 0.41 |
| E3 | 0.59 – 0.73 | 0.70 – 0.83 | 0.92 – 1.08 | 0.99 – 1.16 | 1.36 – 1.48 | 1.01 – 1.07 |
| E4 | 0.51 – 0.63 | 0.60 – 0.73 | 0.81 – 0.97 | 0.96 – 1.14 | 1.27 – 1.41 | 0.92 – 0.99 |
| E5 | **0.99 – 1.16** | **1.26 – 1.43** | **1.44 – 1.61** | **1.52 – 1.70** | **1.91 – 2.04** | **1.53 – 1.61** |
| E6 | **0.79 – 0.95** | **0.98 – 1.14** | **1.18 – 1.35** | **1.31 – 1.49** | **1.76 – 1.89** | **1.31 – 1.39** |

Note: Bold indicates confidence intervals that do not overlap with confidence interval within group (number of trauma types), indicating symptoms of higher severity.

B1: recurrent thoughts of trauma.B2: recurrent dreams of trauma. B3: flashbacks. B4: psychological cue reactivity. B5: physiological cue reactivity. C1: avoidance of thoughts of trauma. C2: avoidance of reminders of trauma. D1: memory impairment. D2: negative beliefs. D3: distorted blame. D4: persistent negative emotional state. D5: diminished interested in activities. D6: feelings of detachment from others. D7: inability to experience positive emotions. E1: irritability or anger. E2: reckless/self destructive behaviour. E3: hypervigilance. E4: exaggerated startle response. E5: difficulty concentrating. E6: sleeping difficulties


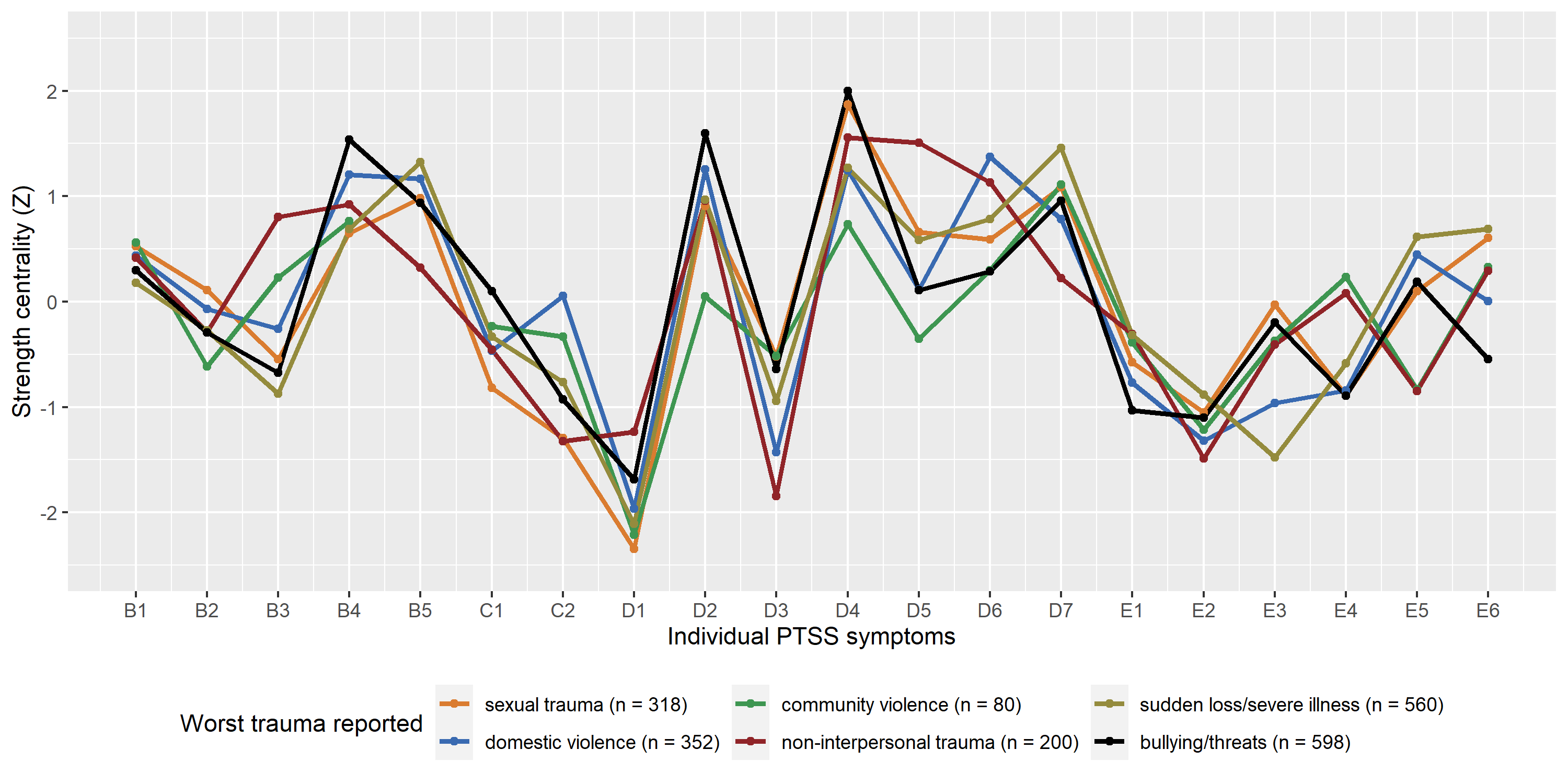


Figure S1. Strength centrality of each of the individuals PTSS symptoms according to worst trauma reported.

B1: recurrent thoughts of trauma.B2: recurrent dreams of trauma. B3: flashbacks. B4: psychological cue reactivity. B5: physiological cue reactivity. C1: avoidance of thoughts of trauma. C2: avoidance of reminders of trauma. D1: memory impairment.D2: negative beliefs. D3: distorted blame. D4: persistent negative emotional state. D5: diminished interested in activities. D6: feelings of detachment from others. D7: inability to experience positive emotions. E1: irritability or anger. E2: reckless/self-destructive behaviour. E3: hypervigilance. E4: exaggerated startle response. E5: difficulty concentrating. E6: sleeping difficulties.
